# Understanding HIV and associated risk factors among religious groups in Zimbabwe

**DOI:** 10.1186/s12889-021-10405-8

**Published:** 2021-02-17

**Authors:** Munyaradzi Mapingure, Zindoga Mukandavire, Innocent Chingombe, Diego Cuadros, Farirai Mutenherwa, Owen Mugurungi, Godfrey Musuka

**Affiliations:** 1 ICAP at Columbia University, Harare, Zimbabwe; 2 Centre for Data Science, Coventry University, Coventry, UK; 3 School of Computing, Electronics and Mathematics, Coventry University, Coventry, UK; 4 University of Cincinnati, Cincinnati, USA; 5 Biomedical Research & Training Institute, Harare, Zimbabwe; 6 Ministry of Health and Child Care, Harare, Zimbabwe

**Keywords:** HIV, Risk factors, Religious groups, Prevention

## Abstract

**Background:**

The influence of religion and belief systems is widely recognized as an important factor in understanding of health risk perception and myths in the general fight against the HIV pandemic. This study compares the understanding of HIV risk factors and utilization of some HIV services among religious groups in Zimbabwe.

**Methods:**

We conducted secondary data statistical analysis to investigate the understanding of HIV and associated risk factors among religious groups in Zimbabwe using 2015–2016 Zimbabwe Demographic and Health Survey (ZDHS) data. We began by investigating associations between understanding of HIV and associated risk factors among religious groups. A multivariate stepwise backward elimination method was carried out to explore factors determining understanding of HIV risk after controlling for confounding factors using the most recent ZDHS data (2015–2016).

**Results:**

The results from the three surveys showed that, in general apostolic sector had low understanding of HIV and associated risk factors compared to other religious groups. Analysis of the 2015–2016 ZDHS data showed that women belonging to the apostolic sector were less likely to know where to get an HIV test odds ratio (OR) and 95% confidence interval, 0.665 (0.503–0.880) and to know that male circumcision reduces HIV transmission OR 0.863 (0.781–0.955). Women from this group had no knowledge that circumcised men can be infected if they do not use condoms OR 0.633 (0.579–0.693), nor that it is possible for a healthy-looking person to have HIV, OR 0.814 (0.719–0.921). They would not buy vegetables from a vendor with HIV OR 0.817 (0.729–0.915) and were less likely to support that HIV positive children should be allowed to attend school with HIV negative children OR 0.804 (0.680–0.950). Similar results were obtained for men in the apostolic sector. These men also did not agree that women were justified to use condoms if the husband has an Sexually Transmitted Infection (STI) OR 0.851 (0.748–0.967).

**Conclusions:**

Our results suggest that apostolic sector lack adequate knowledge of HIV and associated risk factors than other religious groups. Targeting HIV prevention programmes by religious groups could be an efficient approach for controlling HIV in Zimbabwe.

**Supplementary Information:**

The online version contains supplementary material available at 10.1186/s12889-021-10405-8.

## Introduction

Religion has a permeating influence on all aspects of life [1, 2]. Its role in explaining health access and health outcomes has received considerable scholarly debate in Africa [3–5]. The church, as a community, provides a unique platform for informal social interaction as well as formal teaching and regulation, thus shaping individual attitudes towards health-seeking behavior [6]. The influence of religious organizations is widely recognized as an important factor in the fight against the HIV epidemic, particularly in sub-Saharan Africa [7–9]. However, the causal mechanisms that explain the associations between religion and health have largely been inconclusive [10]. On the one hand, there are arguments that support the view that church doctrine; religious beliefs and values have a direct influence on health outcomes [10], while on the other hand, others maintain that the observed differences on health outcomes are not a result of religion itself but rather due to differential access to social and human capital [11, 12]. The effects of religious affiliation on health and their implications for prevention and care programmes therefore require further studying in order to guide the design of effective HIV prevention and mitigation programmes.

**Fig. 1:**
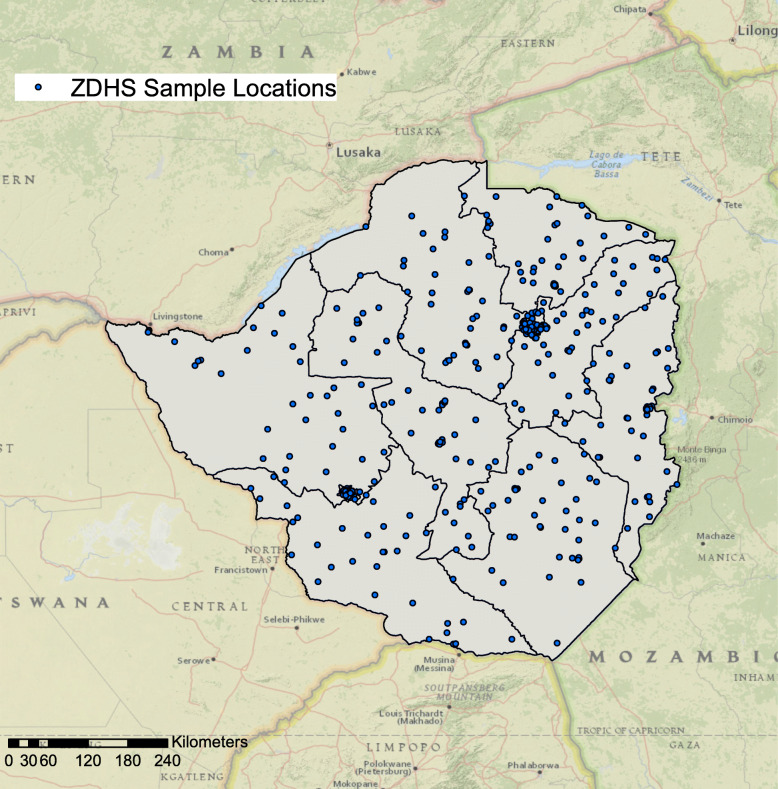
Distribution of main religions by provinces in Zimbabwe. Maps were created using ArcGIS® software by Esri version 10.3 (http://www.esri.com/)

Zimbabwe is predominantly a Christian nation. Approximately, 87% of the population practice Christianity [13] under different church denominations [14]. According to the 2015 Zimbabwe Demographic and Health Survey (ZDHS), Apostolics were identified as the largest religious denomination in the country constituting 38% of the population ages 15–49 years [13], and is the most common religion denomination in most of the provinces in Zimbabwe (Fig. 1). Other religious affiliations in the country were distributed as follows: Pentecostal 22%, Protestant 16%, Roman Catholic 7%, Other Christian 5%, no religion 11%, traditional 2% and Moslem 1% [13]. In spite of the popularity of Christianity in the country there is great variability amongst the different churches and denominations in terms of beliefs, teachings and practices on sexual and health seeking behaviors. For example, while polygamy is not approved in the mainline churches, in particular those with Missionary foundations, it is acceptable in most African “Independent, Initiated, Indigenous, Instituted” Churches (AICs) [15]. The provision and promotion of western health beliefs and medicine, while abhorrent to most AICs, is widely accepted by the mainline churches [14, 15].

Although the last decade has seen the rapid emergence and expansion of Charismatic Evangelical denominations, primarily dominated by the Pentecostal churches, the apostolic churches still have a substantial following [15]. Christians who practice the apostolic ‘brand’ of faith religion are commonly referred to as *Vapositori*, a Shona adaptation of the word “*apostolics*” [4, 5, 16]. A trend analysis suggests that there has been a steady increase on group membership among the apostolics from 20% of the Zimbabwean population in the 1990s [17] to 27% in 2009 [18] and currently at 38% [13]. Forty two percent of the women respondents reported that they belonged to the apostolic sect [13]. Of the several apostolic churches in Zimbabwe, the Johanne Marange and Johanne Masowe apostolic groups command a huge following with members spread across the African continent and beyond [19].

It must be highlighted, however, that the collective term *vapositori* masks the diversity among Christians commonly identified by this name. For example, they differ on theological foundations, are quite varied in terms of their beliefs, values, teachings, regulations and practices. An attempt to profile these churches is documented elsewhere [4] and is beyond the scope of this study. However, for comparative purposes, we adopt a taxonomy reported by Maguranyanga (2011), which categorizes apostolics on a continuum that ranges from the “ultra conservative” or “fundamentalists” to the “semi-conservative” and “liberal” apostolic groups based on their beliefs on uptake of modern medical services. The ultra-conservative apostolics teach against any access and utilization of modern healthcare services and place great emphasis on faith healing through prayers, the use of holy water and stones while the semi-conservative groups neither object nor openly promote use of modern medicine and actively seek healthcare [4]. All these attributes and characteristics are mirrored in the followers’ health seeking practices and attitudes towards conventional medicine.

Most of the available literature on the role of religion in Zimbabwe, in general and the apostolics in particular, on access to health services specifically has focused on small cross-sectional studies within specific geographic areas [20–22]. Consequently, these studies may not have been representative of the group nationally and the other southern African countries where this group exists. For example, a study conducted in Manicaland demonstrated the negative impact of affiliation to an apostolic church to child mortality [20]. Another study showed that mothers belonging to the Apostolic faith were less likely to have used postnatal care services in an urban suburb in Harare [21]. There are few studies that have used population based national data to understand the relationship between religion and access to health in general and among the apostolics in particular, however, they have their fair share of limitations. For example, an analysis of data from the Zimbabwe’s Demographic and Health Survey 2005 [23] showed that apostolic women were at higher risk of HIV infection because they married early. The study however, only controlled for age and did not account for other potential confounding effects. Another, nationally representative study showed that affiliation with the Apostolic faith was a significant risk factor in reducing utilization of maternal and child health services despite reduced costs, accessibility and availability of these services [10]. However, since the primary focus of the study was maternal and child health, the relationship between HIV and its associated risks was not explored and the study did not control for other confounding factors. Given this background, using data from a large nationally representative survey (ZDHS) this study investigates the understanding of the HIV epidemic and associated risk factors for HIV infection among religious groups in Zimbabwe.

## Methods

### Study area and data sources

**Fig. 2:**
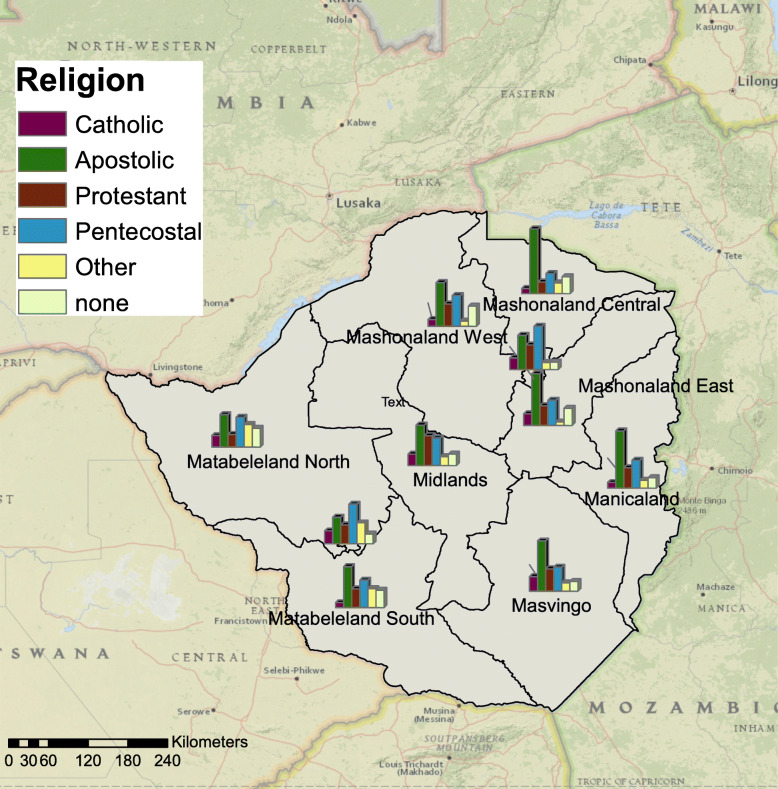
Zimbabwe Demographic and Health Survey (ZDHS) sample locations (blue dots). Maps were created using ArcGIS® software by Esri version 10.3 (http://www.esri.com/)

The ZDHS study area was Zimbabwe which has one of the highest burden of HIV in the world, with approximately 1.2 million persons aged 15–64 years old living with HIV in 2016 [24]. The main source of secondary data for this study was the most recent ZDHS [25]. Figure 2 shows the geographic locations utilized for sample collection.

### Statistical analysis

Firstly, we explored associations between variables using simple chi-square test for categorical variables. Thereafter, multivariate stepwise backward elimination method was used to explore factors significantly associated with the understanding of the HIV infection and associated risk factors comparing apostolic and other religions after controlling for confounding among these factors. Two models were analyzed separately i.e. one for males and the other for females, to explore independent associations for each gender. Briefly, stepwise regression is a method of fitting regression models in which the choice of predictive variables is carried out by an automatic procedure in STATA or other statistical packages. We specifically conducted the backward elimination procedure which involves starting with all candidate variables, testing the deletion of each variable using a chosen model fit criterion, deleting the variable (if any) whose loss gives the most statistically insignificant deterioration of the model fit, and repeating this process until no further variables can be deleted without a statistically significant loss of fit. Additionally, we used the current ZDHS 2015–16 and two previous rounds done in 2010–11 and 2005–06 to conduct some chi-square tests on the HIV knowledge and HIV risk factor variables. We conducted additional analysis on the latest round of DHS as recent data would more useful in shaping the current HIV programming techniques in Zimbabwe,

## Results

### Demographic characteristics for 2015 ZDHS survey

The demographic characteristics for the ZDHS 2015 are presented in Table 1. More than half of the (62% for females and 59% for males) survey participants of the population lived in rural areas. Two thirds, (66% for both sexes) of survey participants had secondary education. The Apostolic Sect was the largest religious group, constituting 40% of women and 30% of all adult males.

**Table 1:** Baseline frequencies for participant characteristics using ZDHS 2015–16 data

Variable	Female Frequency n (%)	Male frequency n (%)
Age group in years		
15–19	2156 (22)	2065 (25)
20–24	1782 (17)	1376 (16)
25–29	1656 (17)	1166 (14)
30–34	1591 (16)	1104 (13)
35–39	1209 (12)	932 (11)
40–44	966 (10)	797 (9)
45–49	595 (6)	578 (7)
50–54 (Men only)		378 (5)
Type of residence		
Urban	4521 (38)	3456 (41)
Rural	5434 (62)	4940 (59)
Highest education level		
None	106 (1)	57 (1)
Primary	2385 (26)	1855 (22)
Secondary	6637 (66)	5524 (66)
Higher	827 (7)	960 (11)
Marital status		
Never in union	2666 (25)	3619 (43)
Married	5700 (59)	4267 (51)
Living with partner	315 (3)	70 (1)
Widowed	430 (4)	66 (1)
Divorced	488 (5)	163 (2)
Separated	356 (4)	211 (3)
Religion		
Traditional	60 (1)	220 (3)
Roman catholic	670 (7)	698 (8)
Protestant	1618 (16)	1272 (15)
Pentecostal	2679 (25)	1551 (18)
Apostolic sect	3829 (42)	2507 (30)
Other Christian	589 (5)	606 (7)
Muslim	30 (0)	53 (1)
None	471 (5)	1479 (18)
Other	9 (0)	10 (0)

### HIV knowledge factors associated with the apostolic sect

The results in Table 2 shows that the apostolic males and females were less likely to have ever heard about HIV when respectively compared to males and females of other religions (*p* < 0.05) and ever tested for HIV. When compared to males and females of other religions the apostolic sect showed lower percentage of knowledge. Comprehensive knowledge on HIV was only 47% among females Apostolics compared to 60% among non Apostolics females. Similarly, comprehensive knowledge on HIV was only 51% among males Apostolics compared to 58% among non Apostolics males.

**Table 2:** Factors associated with the apostolic sector analyzed separately for males and females from ZDHS 2015–16 data

Variable	Females n (%)			Males n (%)		
	Apostolics	Other religion	***P*** value	Apostolics	Other religion	***P*** value
Ever heard about HIV						
No	55 (1.4)	34 (0.6)		27 (1.2)	28 (0.5)	
Yes	3774 (98.6)	6092 (99.4)	0.011	2480 (98.8)	5861 (99.5)	0.002
Ever tested for HIV						
No	805 (21.9)	1085 (17.5)		1016 (42.0)	1873 (32.2)	
Yes	3024 (78.1)	5041 (82.5)	0.006	1491 (58.0)	4016 (67.8)	0.001
Know where to get tested						
No	126 (3.6)	124 (2.3)		150 (6.2)	170 (3.0)	
Yes	496 (96.4)	5947 (97.7)	0.001	2330 (93.8)	5691 (97.0)	0.001
HIV transmission can be reduced by having 1 sexual partner						
No	7282 (7.6)	330 (5.4)		145 (5.8)	251 (4.7)	
Yes	3434 (90.8)	5705 (93.5)		2312 (93.3)	5576 (94.7)	
Don’t know	58 (1.6)	57 (1.1)	0.023	23 (1.0)	34 (0.7)	0.062
Condom use reduces HIV						
No	519 (13.9)	705 (11.8)		300 (11.8)	517 (9.1)	
Yes	3125 (82.1)	5282 (86.0)		2146 (86.6)	5298 (90.1)	
Don’t know	130 (4.0)	105 (2.2)	0.006	34 (1.6)	46 (0.8)	0.001
Men can reduce HIV transmission by being circumcised						
No	672 (18.2)	829 (13.7)		446 (18.3)	925 (15.5)	
Yes	2733 (71.1)	4794 (77.6)		1907 (76.5)	4694 (79.7)	
Don’t know	369 (10.7)	469 (8.6)	0.001	127 (5.3)	242 (4.8)	0.016
Circumcised men who have sex without condom can get HIV/AIDS						
No	981 (26.5)	1029 (17.4)		329 (13.2)	626 (10.5)	
Yes	2210 (56.1)	4357 (68.7)		2016 (81.2)	5011 (85.1)	
Don’t know	583 (17.4)	706 (12.9)	0.001	135 (5.6)	224 (4.4)	0.001
Can get HIV from sharing food						
No	3371 (89.1)	5672 (93.2)		2197 (88.8)	5268 (90.0)	
Yes	312 (8.2)	343 (5.3)		220 (8.6)	490 (8.1)	
Don’t know	91 (2.7)	77 (1.5)	0.001	63 (2.6)	103 (1.9)	0.134
Can get HIV by witchcraft or supernatural means						
No	3456 (91.8)	5696 (93.8)		2279 (92.2)	5403 (92.5)	
Yes	239 (6.0)	306 (4.6)		146 (5.5)	353 (5.6)	
Don’t know	79 (2.2)	90 (1.6)	0.001	55 (2.4)	105 (1.9)	0.442
A healthy person can be HIV infected						
No	604 (15.7)	680 (11.3)		311 (13.1)	580 (10.3)	
Yes	3131 (83.1)	365 (87.8)		2150 (85.9)	5242 (88.9)	
Don’t know	39 (1.2)	47 (0.9)	0.001	19 (1.0)	39 (0.8)	0.005
Would be ashamed if a family member gets infected with HIV						
Disagree	3253 (85.4)	5469 (89.0)		1946 (81.5)	4942 (77.4)	
Agree	511 (14.4)	607 (10.7)		523 (22.2)	895 (16.0)	
Don’t know	10 (0.2)	16 (0.3)	0.001	11 (0.4)	24 (0.56)	0.001
Would buy vegetables from a vendor with HIV						
No	61 (23.4)	968 (16.4)		464 (18.3)	923 (15.3)	
Yes	2888 (76.0)	5093 (82.9)		2002 (81.2)	4879 (83.7)	
Don’t know	25 (0.6)	31 (0.7)	0.001	14 (0.5)	59 (1.0)	0.001
Children with HIV should be allowed to attend school with children without						
No	324 (8.7)	312 (4.6)		245 (9.2)	486 (8.1)	
Yes	3409 (90.1)	5741 (94.6)		2211 (89.9)	5341 (91.2)	
Don’t know	41 (1.2)	39 (0.8)	0.001	24 (0.9)	34 (0.7)	0.201
Wife justified to ask husband to use a condom if he has an STI						
No	509 (12.9)	662 (10.2)		397 (16.0)	670 (12.3)	
Yes	3240 (84.8)	5372 (88.2)		2076 (82.6)	5164 (86.7)	
Don’t know	80 (2.3)	92 (1.6)	0.040	34 (1.4)	55 (1.0)	0.001
Responded circumcised						
No				2196 (88.7)	4861 (84.2)	
Yes				306 (11.2)	1021 (15.7)	
Don’t know				5 (0.2)	7 (0.1)	0.001

### Adjusted regression model results for females

As shown in Table 3, in multivariate stepwise regressing models, compared to women of other religions, women belonging to the Apostolic sect continued to fair negatively in the following items. They are less likely to know where to get an HIV test adjusted odds ratio (aOR) (95% confidence interval [CI]) = 0.665 (0.503–0.880), *p* = 0.004, they are less likely to know that men can reduce their chance of getting HIV by being circumcised, aOR (95%CI) = 0.863 (0.781–0.955), *p* = 0.004. Apostolic women were likely to show misconception that one can get HIV from sharing food OR (95%CI) = 1.203 (1.012–1.429), *p* = 0.036. On a positive note apostolic females had higher rate of HIV testing aOR 1283 (95% CI 1.134–1.451).

**Table 3:** Multivariate stepwise backward elimination model results for females: final model using ZDHS 2015 data

Variable	Adjusted Odds Ratio	95% CI	***P*** Value
Ever tested for HIV	1.283	1.134–1.451	0.001
Know where to get tested	0.665	0.503–0.880	0.004
HIV transmission can be reduced by having 1 sexual partner	0.900	0.765–1.059	0.205
Men can reduce HIV transmission by being circumcised	0.863	0.781–0.955	0.004
Circumcised men who have sex without condom can get HIV/AIDS	0.633	0.579–0.693	0.001
Can get HIV from sharing food	1.203	1.012–1.429	0.036
Can get HIV by witchcraft or supernatural means	1.134	0.943–1.362	0.181
A healthy person can be HIV infected	0.814	0.719–0.921	0.001
Would be ashamed if a family member gets infected with HIV	1.110	0.970–1.270	0.129
Would buy vegetables from a vendor with HIV	0.817	0.729–0.915	0.001
Children with HIV should be allowed to attend school with children without HIV	0.804	0.680–0.950	0.011

### Adjusted regression model results for males

As shown in Table 4, in multivariate stepwise regressing models, compared to men of other religions, men belonging to the apostolic sect were less likely to have been tested for HIV, aOR (5%CI) = 0.825 (0.743–0.916), *p* = 0.001, or know where to get an HIV test, aOR (5%CI) = 0.637 (0.502–0.809), *p* = 0.001. The men were less likely to know that circumcised men who have sex without a condom can get HIV aOR (5%CI) = 0.771 (0.665–0.895), *p* = 0.001. Apostolic men were less likely to be circumcised aOR (5%CI) = 0.773 (0.675–0.885), *p* = 0.001.

**Table 4:** Multivariate stepwise backward elimination model results for males: final model using ZDHS 2015 data

Variable	Adjusted Odds Ratio	95% CI	***P*** Value
Ever tested for HIV	0.825	0.743–0.916	< 0.001
Know where to get tested	0.637	0.502–0.809	< 0.001
Circumcised men who have sex without condom can get HIV/AIDS	0.771	0.665–0.895	0.001
Would be ashamed if a family member gets infected with HIV	0.709	0.616–0.816	0.001
Wife justified to ask husband to use a condom if he has an STI	0.851	0.748–0.967	0.014
Responded circumcised	0.773	0.675–0.885	0.001

### Trend of risk perception

From data analysis results of the three rounds of ZDHS surveys, Apostolics male and females performed poorly in most questionnaire items related to their understanding of HIV and associated risk factors, see supplementary Tables 1a, b, 2a, b, 3 and 4.

With regard to data on previous rounds of ZDHS, we also performed chi-square tests for various factors comparing the apostolic sect and people of other religions using the ZDHS 2010–2011 data (supplementary Table 3) and ZDHS 2005–2006 data (supplementary Table 4).

## Discussion

We conducted a comparative analysis of understanding HIV risk factors among religions groups in Zimbabwe. Our findings support previous findings that Apostolics are lagging behind in terms of their behavior and understanding of HIV issues. Our findings are consistent with previous studies in Zimbabwe which shown that affiliation to an apostolic church has some impact on health seeking behavior, access to health and health outcomes [4, 10, 20, 21]. This association could be explained in one of several ways. First, the church provides social support mechanisms in times of need, which potentially results in improved mental and physical health for church members. Second, the strict church doctrine on sex and sexuality and moral codes of conduct have a strong influence on members sexual practices and on individual and collective HIV risk perceptions [20, 26]. Thirdly, health, disease and illness are perceived to have primarily a spiritual foundation and healing is believed to come from prophets through acts of the Holy Spirit [4]. Any reliance on traditional or western preventative and curative services is therefore viewed as a display of lack of faith [5] and may be condemned and despised by some apostolic groups [4]. It is no wonder that some apostolic sects openly object to the uptake of modern medical health services including immunization and the taking of medicines [4, 27].

Univariate analysis showed that while knowledge of existence of HIV is universal, the percentages of apostolics who has this knowledge is slightly lower. The proportions of ever receiving an HIV test or knowing where HIV testing is done are also low for both males and females. Because Apostolic forms an increasingly large proportion of the people in Zimbabwe [13, 17, 18], targeting these groups with HIV testing messages and access to HIV testing, will certainly increase the first 90 of The Joint United Nations Programme on HIV/AIDS (UNIAIDS’s) 90–90-90 targets. The construct variable for comprehensive knowledge of HIV also shows lack of knowledge among apostolic groups.

The results showed misconception with regards to HIV prevention and modes of transmission among apostolic males and females when compared to other religions. This pose serious challenges with regards to HIV prevention hence the need for targeted information around HIV transmission and prevention. Of note, is their failing to mention that HIV transmission can be reduced by having one HIV negative sexual partner who is faithful. This is also embodied in their religious teaching around having to be fruitful and multiply and of note is that apostolic groupings engage in polygamous marriages. The apostolic males and females are less likely to think that a healthy person may be having HIV infection and may therefore indulge in unsafe sex which would increase their chances of being positive. It is also disturbing to note that both male and female apostolic are less likely to think that a wife is justified to ask her husband to use a condom if he has an STI.

Our analysis also showed that male apostolic struggle with stigma issues and also on how they would relate to an HIV infected person. As previously reported [5] religion affects people’s daily lives by solving social problems, although it creates others. Efforts to reduce stigma and discrimination around the apostolic is warranted.

It is worth noting that in multivariate regression models there are crucial HIV related factors that remained poor among these groups. The main limitation of this this study is that we did not disaggregate data on the apostolic sector as other studies have shown that it is not one homogenous group as captured by the ZDHS survey data but at least three groups: the ultraconservative, the semi-conservative and the liberal Apostolic groups which place varying emphasis on faith healing and the strict adherence to church beliefs against the use of modern medicine. However, our results may be interpreted as generalizing the situation among this group.

## Conclusions

The study results give a more comprehensive information on understanding of HIV and associated risk factors amongst the largest key religious groups in Zimbabwe. These results show that HIV knowledge and understanding is lower in the Apostolic sector compared to other religious groups and the odds of HIV high risk behavior is higher in this sector than other religious groups. This association remained even after adequately controlling for other mediating factors. This conclusion is supported by a number of other studies on health seeking behavior generally amongst the apostolic sect but not specifically referring to HIV prevention and care services [5]. Some studies [4, 5] suggest that “religious teaching and church regulations, of the Apostolic sect groups for example faith healing, negatively shape healthcare-seeking behavior”. It is important to highlight that this study focused on the association between HIV knowledge and religious practices and believes. This association should not be treated as evidence that it is the beliefs and practices of the different religious groups that explain the systematic differences we found, and thus our results should be interpreted with caution.

In order to overcome theological rigidity on health related issues among the apostolic sector, the Ministry of Health and Child Care (MoHCC) and its implementing partners should work more closely with apostolic sector representative organizations to develop enhanced, targeted information, education and communication materials and promotional events to address misinformation, myths and lack of understanding on HIV prevention and care.

## Supplementary Information

**Additional file 1.**

## Data Availability

The data that support the findings of this study are available from the Demographic and Health Surveys (http://www.measuredhs.com) but restrictions apply to the availability of these data, which were used under license for the current study, and so are not publicly available.

## Abbreviations

AICs: African “Independent, Initiated, Indigenous, Instituted” Churches
aOR: Adjusted Odds Ratio
EA: Enumeration Area
HIV: Human Immunodeficiency Virus
IRB: Institutional Review Board
MoHCC: Ministry of Health and Child Care
OR: Odds Ratio
STI: Sexually Transmitted Infection
UNIAIDS: The Joint United Nations Programme on HIV/AIDS
ZDHS: Zimbabwe Demographic and Health Survey
