# Supplementary material for: Understanding HIV and associated risk factors among religious groups in Zimbabwe
Source: BMC Public Health. 2021 Feb 17;21:375. doi: 10.1186/s12889-021-10405-8 (PMC7891154; doi:10.1186/s12889-021-10405-8)
Supplement: Supplementary file 1 — Additional file 1. [file 12889_2021_10405_MOESM1_ESM.docx]

**Supplementary Results**

**Understanding HIV and associated risk factors among the apostolic sect in Zimbabwe: Implications for prevention and care programmes**

Supplementary Table 1a. Comparison of factors across various religious groups using chi-square tests of ZDHS 2015-16 data for females

| **Variable** | **Females n (%)** | | | | | | | | | |
| --- | --- | --- | --- | --- | --- | --- | --- | --- | --- | --- |
|  | **Traditional** | **Roman Catholic** | **Protestant** | **Pentecostal** | **Apostolic** | **Other Christians** | **Muslim** | **None** | **Other** | **P value** |
| Ever heard about HIV  No  Yes | 0 (0.0)  60 (100.0) | 3 (0.8)  667 (99.2) | 7 (0.4)  1611 (99.6) | 12 (0.4)  2667 (99.6) | 55 (1.4)  3774 (98.6) | 8 (1.3)  581 (98.7) | 1 (3.6)  29 (96.4) | 3 (0.9)  468 (99.1) | 0 (0.0)  9 (100.0) | 0.003 |
| Ever tested for HIV  No  Yes | 18 (25.8)  42 (74.2) | 115 (17.8)  555 (82.2) | 308 (18.8)  1310 (81.3) | 457 (16.6)  2222 (83.4) | 805 (21.9)  3024 (78.1) | 118 (21.0)  471 (79.0) | 5 (18.2)  25 (81.8) | 64 (13.8)  407 (86.2) | 0 (0.0)  9 (100.0) | <0.001 |
| Know where to get tested  No  Yes | 3 (6.6)  57 (93.4) | 12 (2.0)  654 (98.0) | 33 (2.6)  1574 (97.4) | 52 (2.1)  2609 (97.9) | 126 (3.6)  3496 (96.4) | 15 (2.2)  564 (97.8) | 1 (6.4)  27 (93.6) | 8 (1.8)  453 (98.3) | 0 (0.0)  9 (100.0) | 0.043 |
| HIV Test Result  Negative  Positive | 54 (90.1)  4 (9.9) | 501 (83.2)  99 (16.9) | 1230 (85.3)  226 (14.7) | 2019 (82.6)  435 (17.5) | 2853 (83.5)  605 (16.5) | 471 (85.2)  90 (14.8) | 15 (67.4)  9 (32.6) | 322 (77.8)  113 (22.2) | 6 (76.4)  2 (23.7) | 0.018 |
| HIV transmission can be reduced by having 1 sexual partner  No  Yes  Don’t know | 5 (7.3)  55 (92.7)  0 (0.0) | 40 (5.6)  623 (93.5)  4 (0.9) | 59 (4.0)  1542 (95.2)  10 (0.9) | 143 (5.3)  2502 (93.9)  22 (0.9) | 282 (7.6)  3434 (90.8)  58 (1.6) | 47 (8.2)  524 (89.9)  10 (1.9) | 1 (6.1)  28 (93.9)  0 (0.0) | 35 (8.0)  422 (89.7)  11 (2.2) | 0 (0.0)  9 (100.0)  0 (0.0) | 0.001 |
| Condom use reduces HIV  No  Yes  Don’t know | 11 (17.9)  47 (78.5)  2 (3.7) | 66 (10.1)  594 (88.4)  7 (1.6) | 177 (11.5)  1408 (86.7)  26 (1.8) | 291 (11.2)  2335 (86.7)  41 (2.2) | 519 (13.9)  3125 (82.1)  130 (4.0) | 92 (16.4)  474 (80.3)  15 (3.4) | 3 (12.1)  25 (84.0)  1 (3.9) | 65 (13.2)  390 (83.8)  13 (3.0) | 0 (0.0)  9 (100.0)  0 (0.0) | <0.001 |
| Men can reduce HIV transmission by being circumcised  No  Yes  Don’t know | 9 (15.0)  47 (79.8)  4 (5.3) | 83 (13.3)  537 (78.1)  47 (8.6) | 213 (12.5)  1264 (78.5)  134 (9.1) | 325 (12.7)  2153 (79.7)  189 (7.7) | 672 (18.2)  2733 (71.1  369 (10.7) | 105 (18.2)  428 (72.1)  48 (9.7) | 6 (21.4)  20 (63.5)  3 (15.1) | 87 (18.7)  337 (70.1)  44 (11.3) | 1 (24.1)  8 (75.9)  0 (0.0) | <0.001 |
| Circumcised men who have sex without condom can get HIV/AIDS  No  Yes  Don’t know | 20 (29.2)  33 (60.7)  7 (10.1) | 110 (18.1)  480 (68.5)  77 (13.4) | 249 (16.0)  1185 (71.4)  177 (12.6) | 402 (15.7)  1953 (71.8)  312 (12.5) | 981 (26.5)  2210 (56.1)  583 (17.4) | 126 (21.3)  391 (66.1)  64 (12.6) | 3 (8.5)  23 (81.2)  3 (10.3) | 115 (25.1)  288 (58.8)  65 (16.1) | 4 (28.6)  4 (47.3)  1 (24.1) | <0.001 |
| Can get HIV from sharing food  No  Yes  Don’t know | 59 (98.9)  1 (1.1)  0 (0) | 626 (93.1)  34 (5.5)  7 (1.4) | 1514 (94.0)  84 (5.2)  13 (0.8) | 2507 (94.2)  130 (4.3)  30 (1.5) | 3371 (89.1)  312 (8.2)  91 (2.7) | 529 (91.7)  45 (7.0)  7 (1.3) | 25 (88.3)  1 (3.5)  3 (8.2) | 404 (86.8)  47 (9.2)  17 (4.0) | 8 (90.8)  1 (9.2)  0 (0) | <0.001 |
| Can get HIV by witchcraft or supernatural means  No  Yes  Don’t know | 56 (95.6)  1 (1.0)  3 (3.4) | 632 (94.6)  26 (4.2)  9 (1.3) | 1529 (95.3)  58 (3.2)  24 (1.5) | 2496 (93.7)  140 (4.9)  31 (1.4) | 3456 (91.8)  239 (6.0)  79 (2.2) | 534 (93.2)  38 (5.6)  9 (1.3) | 23 (82.3)  4 (9.9)  2 (7.8) | 417 (89.8)  39 (7.5)  12 (2.8) | 9 (100.0)  0 (0.0)  0 (0.0) | <0.001 |
| A healthy person can be HIV infected  No  Yes  Don’t know | 12 (17.6)  48 (82.4)  0 (0.0) | 67 (11.5)  595 (87.6)  5 (1.0) | 153 (10.0)  1446 (89.2)  12 (0.8) | 267 (9.8)  2389 (89.8)  11 (0.4) | 604 (15.7)  3131 (83.1)  39 (1.2) | 90 (14.0)  482 (84.2)  9 (1.9) | 6 (22.2)  22 (73.9)  1 (3.9) | 85 (18.6)  374 (79.3)  9 (2.2) | 0 (0.0)  9 (100.0)  0 (0.0) | <0.001 |
| Would be ashamed if a family member gets infected with HIV Disagree  Agree  Don’t know | 50 (84.0)  10 (16.0)  0 (0.0) | 608 (90.6)  58 (9.2)  1 (0.2) | 1450 (89.1)  156 (10.7)  5 (0.2) | 2389 (88.7)  272 (11.0)  6 (0.3) | 3253 (85.4)  511 (14.5)  10 (0.2) | 525 (90.0)  55 (9.8)  1 (0.2) | 27 (91.5)  1 (4.6)  1 (3.9) | 412 (87.6)  54 (12.0)  2 (0.4) | 8 (89.7)  1 (10.3)  0 (0.0) | <0.001 |
| Would buy vegetables from a vendor with HIV  No  Yes  Don’t know | 14 (26.2)  45 (72.3)  1 (1.5) | 103 (17.4)  560 (81.6)  4 (1.0) | 232 (14.7)  1373 (84.9)  6 (0.4) | 390 (15.0)  2261 (84.1)  16 (0.9) | 861 (23.4)  2888 (76.0)  25 (0.6) | 124 (20.5)  454 (79.1)  3 (0.4) | 5 (17.1)  24 (82.9)  0 (0.0) | 98 (22.9)  369 (77.0)  1 (0.1) | 2 (12.3)  7 (87.7)  0 (0.0) | <0.001 |
| Children with HIV should be allowed to attend school with children without  No  Yes  Don’t know | 3 (4.5)  57 (95.6)  0 (0.0) | 28 (4.3)  631 (94.3)  8 (1.4) | 71 (4.0)  1533 (95.5)  7 (0.6) | 125 (4.1)  2531 (95.4)  11 (0.5) | 324 (8.7)  3409 (90.0)  41 (1.2) | 59 (8.8)  517 (90.1)  5 (1.1) | 0 (0.0)  28 (93.9)  1 (6.1) | 24 (5.8)  437 (92.5)  7 (1.7) | 2 (32.4)  7 (67.6)  0 (0.0) | <0.001 |
| Wife is justified to ask husband to use a condom if he has an STI  No  Yes  Don’t know | 8 (12.9)  51 (86.1)  1 (1.0) | 62 (10.0)  600 (88.7)  8 (1.3) | 153 (8.9)  1443 (89.6)  22 (1.5) | 257 (8.9)  2380 (89.5)  42 (1.7) | 509 (12.9)  3240 (84.8)  80 (2.3) | 100 (14.3)  482 (84.5)  7 (1.2) | 4 (13.2)  25 (81.0)  1 (5.9) | 76 (16.5)  385 (81.1)  10 (2.4) | 2 (34.5)  6 (62.5)  1 (3.1) | <0.001 |

Supplementary Table 1b. Assigning ranks for various factors for six religious groups using the results in table 1a for females

| **Variable** | **Roman Catholic** | **Protestant** | **Pentecostal** | **Apostolic** | **Other Christians** | **None** |
| --- | --- | --- | --- | --- | --- | --- |
| Ever heard about HIV | 4 | 1 | 2 | 6 | 3 | 5 |
| Ever tested for HIV | 1 | 3 | 2 | 6 | 5 | 4 |
| Know where to get tested | 3 | 1 | 2 | 6 | 4 | 5 |
| HIV Status Obtained from ZDHS | 4 | 1 | 5 | 3 | 2 | 6 |
| HIV transmission can be reduced by having 1 sexual partner | 1 | 3 | 2 | 5 | 6 | 4 |
| Condom use reduces HIV | 1 | 3 | 2 | 6 | 5 | 4 |
| Men can reduce HIV transmission by being circumcised | 3 | 2 | 1 | 5 | 4 | 6 |
| Circumcised men who have sex without condom can get HIV/AIDS | 1 | 4 | 2 | 5 | 3 | 6 |
| Can get HIV from sharing food | 3 | 2 | 1 | 4 | 5 | 6 |
| Can get HIV by witchcraft or supernatural means | 1 | 2 | 3 | 5 | 3 | 6 |
| A healthy person can be HIV infected | 1 | 3 | 2 | 6 | 5 | 4 |
| Would be ashamed if a family member gets infected with HIV | 3 | 4 | 1 | 6 | 5 | 2 |
| Would buy vegetables from a vendor with HIV | 2 | 3 | 1 | 4 | 6 | 5 |
| Children with HIV should be allowed to attend school with children without | 3 | 1 | 2 | 5 | 4 | 6 |
| Wife is justified to ask husband to use a condom if he has an STI | 1 | 3 | 2 | 5 | 4 | 6 |
| Total score | 32 | 36 | 30 | 77 | 64 | 75 |

Supplementary Table 2a. Comparison of factors across various religious groups using chi-square tests of ZDHS 2015-16 data for males

| **Variable** | **Males n (%)** | | | | | | | | | |
| --- | --- | --- | --- | --- | --- | --- | --- | --- | --- | --- |
|  | **Traditional** | **Roman Catholic** | **Protestant** | **Pentecostal** | **Apostolic** | **Other Christian** | **Muslim** | **None** | **Other** | **P value** |
| Ever heard about HIV  No  Yes | 1 (0.3)  219 (99.7) | 3 (0.7)  695 (99.3) | 1 (0.1)  1271 (99.9) | 5 (0.3)  1546 (99.7) | 27 (1.2)  2480 (98.8) | 3 (0.5)  603 (99.6) | 0 (0.0)  53 (100.0) | 15 (1.1)  1464 (98.9) | 0 (0.0)  10 (100.0) | 0.014 |
| Ever tested for HIV  No  Yes | 70 (31.6)  150 (68.4) | 189 (29.2)  509 (70.8) | 399 (31.9)  873 (68.1) | 465 (30.6)  1086 (69.4) | 1016 (42.0)  1491 (58.0) | 213 (36.1)  393 (63.9) | 14 (26.0)  39 (74.0) | 520 (34.5)  959 (65.5) | 3 (23.3)  7 (76.7) | <0.001 |
| Know where to get tested  No  Yes | 4 (2.3)  215 (97.7) | 20 (3.2)  675 (96.8) | 29 (2.6)  1242 (97.4) | 44 (2.7)  1502 (97.3) | 150 (6.2)  2330 (93.8) | 19 (3.5)  584 (96.5) | 1 (2.8)  52 (97.2) | 53 (3.7)  1411 (96.3) | 0 (0.0)  10 (100.0) | <0.001 |
| HIV Status  Negative  Positive | 156 (81.5)  42 (18.5) | 528 (87.3)  80 (12.7) | 1008 (90.8)  116 (9.2) | 1230 (90.7)  133 (9.3) | 1937 (89.6)  241 (10.4) | 487 (89.0)  62 (11.0) | 37 (76.9)  10 (23.1) | 1142 (85.7)  203 (14.3) | 6 (72.2)  2 (27.8) | <0.001 |
| HIV transmission can be reduced by having 1 sexual partner  No  Yes  Don’t know | 16 (8.7)  202 (91.1)  1 (0.2) | 19 (3.4)  675 (96.3)  1 (0.3) | 52 (4.5)  1216 (95.2)  3 (0.3) | 58 (3.8)  1484 (95.9)  4 (0.3) | 145 (5.8)  2312 (93.3)  23 (1.0) | 34 (5.9)  562 (93.0)  7 (1.1) | 3 (7.5)  50 (92.5)  0 (.0) | 68 (5.0)  1379 (93.6)  17 (1.4) | 1 (20.8)  8 (68.4)  1 (10.8) | <0.001 |
| Condom use reduces HIV  No  Yes  Don’t know | 15 (7.1)  203 (91.3)  1 (1.6) | 55 (7.8)  639 (92.1)  1 (0.1) | 107 (9.0)  1156 (90.4)  8 (0.6) | 109 (7.4)  1424 (91.8)  13 (0.9) | 300 (11.8)  2146 (86.6)  34 (1.6) | 66 (11.6)  530 (87.3)  7 (1.1) | 3 (6.4)  50 (93.6)  0 (0.0) | 161 (11.0)  1287 (87.8)  16 (1.2) | 1 (6.6)  9 (93.5)  0 (0.0) | 0.006 |
| Men can reduce HIV transmission by being circumcised  No  Yes  Don’t know | 25 (10.3)  179 (78.5)  15 (11.2) | 113 (16.4)  562 (80.9)  20 (2.7) | 188 (15.0)  1039 (81.3)  44 (3.7) | 216 (13.4)  1281 (82.3)  49 (4.2) | 446 (18.3)  1907 (76.5)  127 (5.3) | 116 (19.0)  472 (78.6)  15 (2.4) | 6 (9.2)  47 (90.8)  0 (0.0) | 259 (17.5)  1106 (75.2)  99 (7.4) | 2 (29.8)  8 (70.2)  0 (0.0) | <0.001 |
| Circumcised men who have sex without condom can get HIV/AIDS  No  Yes  Don’t know | 33 (15.0)  173 (77.5)  13 (7.55) | 53 (8.0)  631 (90.6)  11 (1.4) | 122 (10.3)  1108 (85.8)  41 (3.83) | 127 (7.8)  1378 (89.1)  41 (3.05) | 329 (13.2)  2016 (81.2)  135 (5.62) | 72 (11.5)  515 (85.9)  16 (2.65) | 5 (9.9)  47 (87.4)  1 (2.75) | 212 (13.6)  1151 (78.6)  101 (7.76) | 2 (13.7)  8 (86.3)  0 (0.0) | <0.001 |
| Can get HIV from sharing food  No  Yes  Don’t know | 188 (85.8)  24 (11.7)  7 (2.6) | 631 (90.6)  51 (7.4)  13 (2.0) | 1172 (92.4)  77 (5.7)  22 (1.9) | 1444 (93.2)  89 (5.8)  13 (1.0) | 2197 (88.8)  220 (8.6)  63 (2.6) | 519 (87.1)  73 (11.0)  11 (1.9) | 51 (95.9)  2 (4.1)  0 (0.0) | 1254 (85.8)  174 (11.5)  36 (2.7) | 9 (89.2)  0 (0.0)  1 (10.8) | <0.001 |
| Can get HIV by witchcraft or supernatural means  No  Yes  Don’t know | 199 (91.4)  17 (6.9)  3 (1.7) | 647 (94.1)  34 (4.5)  14 (1.4) | 1181 (92.8)  57 (4.5)  33 (2.7) | 1432 (92.6)  94 (5.7)  20 (1.7) | 2279 (92.2)  146 (5.5)  55 (2.4) | 556 (92.6)  37 (5.8)  10 (1.6) | 50 (94.6)  2 (3.2)  1 (2.2) | 1329 (91.4)  111 (6.8)  24 (1.8) | 9 (93.5)  1 (6.6)  0 (0.0) | 0.513 |
| A healthy person can be HIV infected  No  Yes  Don’t know | 12 (7.5)  204 (91.3)  3 (1.2) | 53 (7.0)  638 (92.4)  4 (0.6) | 125 (11.2)  1140 (88.2)  6 (0.6) | 130 (9.1)  1409 (90.3)  7 (0.6) | 311 (13.1)  2150 (85.9)  19 (1.0) | 74 (12.9)  523 (86.0)  6 (1.1) | 5 (10.2)  48 (89.8)  0 (0.0) | 180 (12.0)  1271 (87.0)  13 (1.0) | 1 (9.3)  9 (90.7)  0 (0.0) | 0.023 |
| Would be ashamed if a family member gets infected with HIV  Disagree  Agree  Don’t know | 184 (83.0)  35 (17.0)  0 (0.0) | 594 (83.8)  93 (14.6)  8 (1.7) | 1066 (82.7)  201 (17.0)  4 (0.3) | 1318 (84.0)  225 (15.8)  3 (0.2) | 1946 (77.4)  523 (22.2)  11 (0.4) | 490 (82.1)  111 (17.7)  2 (0.3) | 47 (89.7)  6 (10.3)  0 (0.0) | 1236 (83.9)  221 (15.6)  7 (0.6) | 7 (74.0)  3 (26.1)  0 (0.0) | <0.001 |
| Would buy vegetables from a vendor with HIV  No  Yes  Don’t know | 29 (12.9)  185 (85.5)  5 (1.5) | 95 (12.6)  590 (85.7)  10 (1.7) | 177 (14.4)  1084 (84.7)  10 (0.8) | 189 (12.3)  1341 (86.5)  16 (1.2) | 464 (18.3)  2002 (81.2)  14 (0.5) | 129 (20.3)  470 (79.1)  4 (0.6) | 9 (13.5)  44 (86.5)  0 (0.0) | 292 (18.9)  1159 (80.3)  13 (0.8) | 3 (26.1)  6 (53.1)  1 (20.8) | <0.001 |
| Children with HIV should be allowed to attend school with children without  No  Yes  Don’t know | 20 (9.7)  198 (89.8)  1 (0.5) | 51 (7.4)  641 (92.0)  3 (0.6) | 76 (6.5)  1192 (93.4)  3 (0.2) | 92 (6.4)  1449 (93.3)  5 (0.4) | 245 (9.3)  2211 (89.9)  24 (0.9) | 62 (8.9)  535 (90.0)  6 (1.1) | 4 (6.8)  49 (93.2)  0 (0.0) | 180 (11.3)  1268 (87.4)  16 (1.4) | 1 (10.8)  9 (89.2)  0 (0.0) | <0.001 |
| Wife is justified to ask husband to use a condom if he has an STI  No  Yes  Don’t know | 40 (21.0)  178 (77.9)  2 (1.0) | 66 (9.6)  628 (89.8)  4 (0.7) | 120 (10.0)  1142 (89.1)  10 (1.0) | 147 (10.2)  1394 (89.2)  10 (0.6) | 397 (16.0)  2076 (82.6)  34 (1.4) | 63 (10.9)  537 (87.9)  6 (1.2) | 4 (7.8)  49 (92.2)  0 (0.0) | 230 (17.0)  1226 (81.4)  23 (1.6) | 0 (0.0)  10 (100.0)  0 (0.0) | <0.001 |
| Responded circumcised  No  Yes  Don’t know | 198 (89.2)  21 (10.3)  1 (0.5) | 573 (84.9)  123 (14.9)  2 (0.2) | 1057 (85.1)  214 (14.8)  1 (0.1) | 1252 (82.6)  298 (17.3)  1 (0.0) | 2196 (88.7)  306 (11.2)  5 (0.2) | 473 (78.8)  132 (21.1)  1 (0.1) | 16 (32.0)  37 (68.0)  0 (0.0) | 1283 (87.8)  195 (12.1)  1 (0.0) | 9 (94.0)  1 (6.0)  0 (0.0) | <0.001 |

Table 2b. Assigning ranks for various factors for six religious groups using the results in table 2a for males

| **Variable** | **Roman Catholic** | **Protestant** | **Pentecostal** | **Apostolic** | **Other Christians** | **None** |
| --- | --- | --- | --- | --- | --- | --- |
| Ever heard about HIV | 4 | 1 | 2 | 6 | 3 | 5 |
| Ever tested for HIV | 1 | 3 | 2 | 6 | 5 | 4 |
| Know where to get tested | 3 | 1 | 2 | 6 | 4 | 5 |
| HIV test result | 6 | 1 | 2 | 3 | 4 | 5 |
| HIV transmission can be reduced by having 1 sexual partner | 1 | 3 | 2 | 5 | 6 | 4 |
| Condom use reduces HIV | 1 | 3 | 2 | 6 | 5 | 4 |
| Men can reduce HIV transmission by being circumcised | 3 | 2 | 1 | 5 | 4 | 6 |
| Circumcised men who have sex without condom can get HIV/AIDS | 1 | 4 | 2 | 5 | 3 | 6 |
| Can get HIV from sharing food | 3 | 2 | 1 | 4 | 5 | 6 |
| Can get HIV by witchcraft or supernatural means | 1 | 2 | 3 | 5 | 3 | 6 |
| A healthy person can be HIV infected | 1 | 3 | 2 | 6 | 5 | 4 |
| Would be ashamed if a family member gets infected with HIV | 3 | 4 | 1 | 6 | 5 | 2 |
| Would buy vegetables from a vendor with HIV | 1 | 3 | 2 | 4 | 6 | 5 |
| Children with HIV should be allowed to attend school with children without | 3 | 1 | 2 | 5 | 4 | 6 |
| Wife is justified to ask husband to use a condom if he has an STI | 1 | 3 | 3 | 5 | 4 | 6 |
| Responded circumcised | 3 | 4 | 2 | 6 | 1 | 5 |
| Total score | 36 | 40 | 31 | 83 | 67 | 79 |

**Supplementary table 3. Factors associated with the apostolics religion analyzed separately for males and females using chi-square test for proportions using ZDHS 2010-11 data**

| **Variable** | **Females n (%)** | | | **Males n (%)** | | |
| --- | --- | --- | --- | --- | --- | --- |
|  | **Apostolics** | **Other religions** | **P value** | **Apostolics** | **Other religions** | **P value** |
| Ever heard about HIV  No  Yes | 81 (2.1)  3315 (97.9) | 142 (2.2)  5633 (97.8) | 0.731 | 47 (2.1)  1978 (97.9) | 97 (1.4)  5358 (98.6) | 0.040 |
| Ever tested for HIV  No  Yes | 1282 (39.0)  2033 (61.0) | 2167 (38.9)  3466 (61.1) | 0.877 | 1333 (66.0)  692 (34.0) | 3337 (59.7)  2118 (40.3) | 0.001 |
| Know where to get tested  No  Yes | 277 (9.0)  3038 (91.0) | 337 (6.3)  5296 (93.7) | 0.001 | 278 (13.1)  1700 (86.9) | 559 (9.3)  4799 (90.7) | 0.001 |
| HIV transmission can be reduced by having 1 sexual partner  No  Yes  Don’t know | 218 (6.6)  2996 (90.4)  101 (3.0) | 316 (5.5)  5211 (92.8)  106 (1.7) | 0.001 | 130 (6.2)  1811 (92.2)  37 (1.6) | 364 (5.9)  4919 (92.8)  75 (1.3) | 0.600 |
| Condom use reduces HIV  No  Yes  Don’t know | 465 (14.6)  2702 (80.8)  148 (4.6) | 673 (12.4)  4760 (83.9)  200 (3.7) | 0.003 | 322 (16.2)  1585 (80.2)  71 (3.6) | 729 (12.8)  4532 (85.3)  97 (1.9) | 0.001 |
| Can get HIV from sharing food  No  Yes  Don’t know | 2932 (88.7)  299 (8.7)  84 (2.6) | 5077 (90.7)  433 (7.3)  123 (2.0) | 0.020 | 1673 (86.1)  249 (11.4)  56 (2.5) | 4647 (87.8)  532 (9.1)  179 (3.1) | 0.013 |
| Can get HIV by witchcraft or supernatural means  No  Yes  Don’t know | 3047 (91.9)  182 (5.2)  86 (2.9) | 5311 (94.6)  223 (3.7)  99 (1.7) | 0.001 | 1801 (91.3)  118 (5.7)  59 (3.0) | 4922 (92.2)  259 (4.5)  177 (3.3) | 0.148 |
| A healthy person can be HIV infected  No  Yes  Don’t know | 362 (10.7)  2890 (87.2)  63 (2.1) | 458 (8.0)  5067 (90.2)  108 (1.8) | 0.001 | 232 (12.2)  1715 (86.2)  31 (1.6) | 558 (10.0)  4740 (89.0)  60 (1.0) | 0.005 |
| Would want HIV infection in family to remain secret  Disagree  Agree  Don’t know | 1761 (52.4)  1483 (45.6)  71 (2.0) | 3189 (56.3)  2218 (39.6)  226 (4.1) | 0.001 | 1115 (55.0)  807 (42.0)  56 (3.0) | 3052 (55.9)  2047 (39.4)  259 (4.7) | 0.005 |
| Would buy vegetables from a vendor with HIV  No  Yes  Don’t know | 870 (26.5)  2422 (72.8)  23 (0.7) | 1059 (18.1)  4523 (81.0)  51 (0.9) | 0.001 | 478 (22.2)  1483 (76.9)  17 (0.9) | 1058 (18.0)  4261 (81.3)  39 (0.7) | 0.001 |
| A male teacher with aids virus should be allowed to continue teaching  No  Yes  Don’t know | 449 (13.3)  2751 (83.0)  115 (3.7) | 495 (8.5)  4967 (88.7)  171 (2.8) | 0.001 | 381 (18.0)  1528 (78.8)  69 (3.2) | 768 (13.0)  4421 (84.2)  169 (2.8) | 0.001 |
| Wife justified to ask husband to use a condom if he has an STI  No  Yes  Don’t know | 579 (17.7)  2711 (79.3)  106 (3.0) | 887 (16.0)  4697 (80.9)  191 (3.1) | 0.153 | 352 (16.0)  1618 (81.2)  55 (2.8) | 825 (13.6)  4526 (84.6)  104 (1.8) | 0.002 |
| Responded circumcised  No  Yes  Don’t know |  |  |  | 1827 (90.7)  173 (8.2)  25 (1.1) | 4898 (89.9)  523 (9.5)  34 (0.6) | 0.023 |

**Supplementary table 4. Factors associated with the apostolics religion analyzed separately for males and females using chi-square test for proportions using ZDHS 2005-6 data**

| **Variable** | **Females n (%)** | | | **Males n (%)** | | |
| --- | --- | --- | --- | --- | --- | --- |
|  | **Apostolics** | **Other religions** | **P value** | **Apostolics** | **Other religions** | **P value** |
| Ever heard about HIV  No  Yes | 80 (2.7)  2592 (97.3) | 134 (1.8)  6099 (98.2) | 0.022 | 24 (1.3)  1579 (98.7) | 41 (0.6)  5529 (99.4) | 0.004 |
| Ever tested for HIV  No  Yes | 2034 (76.8)  615 (23.2) | 4537 (72.8)  1661 (27.2) | 0.001 | 1342 (83.9)  237 (16.1) | 4506 (80.5)  1022 (19.5) | 0.013 |
| Know where to get tested  No  Yes | 503 (21.3)  1783 (78.7) | 775 (12.8)  4791 (87.2) | 0.001 | 497 (29.1)  1081 (70.9) | 1452 (23.9)  4072 (76.1) | 0.001 |
| HIV transmission can be reduced by having 1 sexual partner  No  Yes  Don’t know | 415 (16.4)  2097 (80.9)  77 (2.7) | 944 (15.0)  5033 (83.3)  118 (1.7) | 0.013 | 181 (11.9)  1383 (87.4)  15 (0.8) | 737 (14.6)  4751 (84.9)  34 (0.6) | 0.032 |
| Condom use reduces HIV  No  Yes  Don’t know | 498 (19.5)  1944 (75.7)  146 (4.8) | 1151 (18.1)  4700 (78.2)  240 (3.7) | 0.032 | 275 (17.3)  1271 (80.7)  32 (2.0) | 915 (16.3)  4530 (82.4  74 (1.3) | 0.118 |
| Can get HIV from sharing food  No  Yes  Don’t know | 2066 (80.1)  403 (15.8)  122 (4.1) | 5178 (86.0)  693 (10.5)  219 (3.5) | 0.001 | 1330 (84.3)  204 (12.7)  44 (3.0) | 4734 (86.1)  648 (11.3)  141 (2.7) | 0.393 |
| Can get HIV by witchcraft or supernatural means  No  Yes  Don’t know | 2251 (87.1)  229 (9.1)  106 (3.8) | 5477 (89.9)  411 (6.7)  206 (3.4) | 0.004 | 1445 (91.7)  104 (6.5)  29 (1.8) | 5022 (90.7)  395 (7.2)  105 (2.1) | 0.493 |
| A healthy person can be HIV infected  No  Yes  Don’t know | 323 (13.3)  2191 (84.0)  74 (2.7) | 508 (8.2)  5422 (89.5)  152 (2.3) | 0.001 | 147 (8.5)  1417 (90.7)  15 (0.8) | 379 (6.7)  5104 (92.6)  45 (0.8) | 0.066 |
| Would buy vegetables from a vendor with HIV  No  Yes  Don’t know | 1308 (49.0)  1260 (50.2)  21 (0.8) | 2482 (39.8)  3573 (59.6)  39 (0.6) | 0.001 | 556 (34.9)  1017 (64.7)  6 (0.4) | 1832 (31.6)  3661 (67.9)  30 (0.5) | 0.105 |
| Male teacher with aids virus should be allowed to continue teaching  No  Yes  Don’t know | 828 (31.9)  1649 (63.6)  113 (4.6) | 1374 (21.9)  4502 (74.8)  218 (3.3) | 0.001 | 442 (26.3)  1095 (71.0)  41 (2.7) | 1312 (22.6)  4103 (75.4)  113 (2.0) | 0.016 |
| Wife justified to ask husband to use a condom if he has an STI  No  Yes  Don’t know | 444 (17.9)  2093 (77.9)  119 (4.2) | 676 (11.1)  5285 (85.6)  234 (3.3) | 0.001 | 217 (13.2)  1325 (84.6)  39 (2.2) | 651 (11.6)  4772 (86.9)  96 (1.5) | 0.053 |
| Responded circumcised  No  Yes |  |  |  | 1440 (89.8)  161 (10.2) | 4964 (89.4)  586 (10.6) | 0.728 |
